# Response characteristics of root to moisture change at seedling stage of *Kengyilia hirsuta*

**DOI:** 10.3389/fpls.2022.1052791

**Published:** 2023-01-06

**Authors:** Xueyao Chen, Youjun Chen, Wei Zhang, Wenlu Zhang, Hui Wang, Qingping Zhou

**Affiliations:** ^1^ Sichuan Zoige Alpine Wetland Ecosystem National Observation and Research Station, Southwest Minzu University, Chengdu, China; ^2^ Institute of the Qinghai-Tibetan Plateau, Southwest Minzu University, Chengdu, China

**Keywords:** *Kengyilia hirsuta*, seedling stage, soil moisture, root architecture, root physiological

## Abstract

*Kengyilia hirsuta* is an important pioneer plant distributed on the desertified grassland of the Qinghai-Tibet Plateau. It has strong adaptability to alpine desert habitats, so it can be used as a sand-fixing plant on sandy alpine land. To study the response mechanisms of root morphological and physiological characteristics of *K. hirsuta* to sandy soil moisture, 10%, 25% and 40% moisture levels were set up through potted weighing water control method. The biomass, root-shoot ratio, root architecture parameters, and biochemical parameters malondialdehyde, free proline, soluble protein, indole-3-acetic acid, abscisic acid, cytokinin, gibberellin, relative conductivity and antioxidant enzyme activities were measured in the trefoil stage, and the response mechanisms of roots at different moisture levels were analyzed. The results showed that with the increase of soil moisture, root morphological indexes such as root biomass, total root length, total root volume and total root surface increased, while the root topological index decreased continuously. The malondialdehyde content, relative conductivity, superoxide dismutase activity, peroxidase activity, catalase activity, free proline content, soluble protein content, abscisic acid content and cytokinin content at the 25% and 40% moisture levels were significantly decreased compared with the 10% level (*P<* 0.05). Thus, the root growth of *K. hirsuta* was restricted by the 10% moisture level, but supported by the 25% and 40% moisture levels. An artificial neural network revealed that total root length, total root surface area, root link average length, relative conductivity, soluble protein, free proline and moisture level were the key factors affecting root development. These research results could contribute to future agricultural sustainability.

## 1 Introduction

The alpine grassland of northwest Sichuan located in the southeastern margin of the Qinghai-Tibet Plateau has an important ecological function and is a major water source, which is of great significance to the ecological security of the lower reaches of the Yangtze and Yellow Rivers (Lu et al., 2004; Yu et al., 2011). In recent years, with the increasing population and the demands of social development, which have exerted great pressure on desertified areas, the ecological system has been disturbed and natural resources have been depleted in Northwest China. Since the 1960s, the average annual growth rate of the dune area in the northwest of Sichuan has reached 3.44% (Wei et al., 2010), affecting the development of animal husbandry and leading to the deterioration of the ecological environment (Gou et al., 2019). Against the background of the severe expansion of grassland desertification, choosing suitable herbage species as sand-fixing materials is the main way to achieve good results in artificial restoration of desertified grassland (Chen et al., 2019). Therefore, the selection of pioneer plants with poor-soil and drought tolerance is important to maintain a stable ecosystem.

*Kengyilia hirsuta* is one of the main pioneer grass species distributed on the desertified grassland of the Qinghai-Tibet Plateau, and has strong adaptability to alpine desert habitats, with drought and wind erosion resistance characteristics (Ren, 2016), so it can be used as a forage resource and sand-fixing plant on sandy alpine land. The adaptability of *K. hirsuta* to sandy land mainly depends on the growth and development of its root system, and this trait is co-regulated by internal genetic factors and external environmental factors (Chen et al., 2020).

Soil moisture is the key factor restricting plant growth and development, and the most important factor affecting vegetation restoration in desertified grassland (Knapp et al., 2015). When the soil moisture changes, the root system is the first part affected in desert species (Schiefelbein and Benfey, 1991). Morphological and physical characteristics are the main factors that respond to the complicated environment of desertified grassland, and the biomass, root-shoot ratio, root architecture and root physiology of a plant change under different soil moisture levels (Merchan et al., 2010).

Previous studies have shown that the biomass of plants is the cumulative product of photosynthesis and determined by their photosynthetic capacity (Hester et al., 2001). Under drought stress conditions, aboveground biomass accumulation moves to underground parts, which adapt to the variable environment by changing the root-shoot ratio, enhancing the plant’s ability to resist drought stress (Wang et al., 2016). Root architecture determines the distribution of the root system in the soil space, including root morphology, fractal characteristics and topological structure (Su et al., 2018), and the root system can respond to changes in the external environment through changes of root architecture; for example, total root length, root surface area and root volume are basic indexes closely related to soil moisture (Zeng et al., 2013). Besides, average root diameter, root link average length, number of root tips and number of forks are also closely related to the development of roots in different moisture environments. Where, root link average length is the average length between all two branches of the root system (Tang et al., 2020). Fractal abundance and the fractal dimension are important characteristics of root fractal structure, and the root fractal dimension can be used to study the degree of branching and development, while the fractal abundance is closely related to the extension range and resource competitiveness of roots (Yang and Luo, 1994; Yang et al., 2009). As part of the root system architecture, the topological structure determines the spatial distribution of roots in the soil and affects the nutrient absorption and fixation capacity of roots (Berntson, 1997). The closer the topology index (TI) is to 0.5, the closer the root branching habit is to dichotomous branching, while the closer TI is to 1, the more the root branching habit is similar to herringbone branching (Shan et al., 2012). Under drought stress conditions, root development will be affected to some extent, the fractal dimension and fractal abundance of roots will change, and the root branching mode will tend toward herringbone branching, which is an adaptive strategy for plant roots to expand their space and effectively utilize water and nutrients (Yang et al., 2018).

Root physiology changes with the change of soil moisture, which ultimately affects the root morphological structure (Li et al., 2001). Under conditions of soil moisture deficit, the number of free radicals in plant cells rises, which aggravates membrane lipid peroxidation and affects membrane permeability, resulting in the accumulation of malondialdehyde (MDA) (Lorenz et al., 2011; Zhao et al., 2011), which is a harmful peroxidation product that increases of relative conductivity (RC) (Harfouche et al., 2014). When subjected to drought stress, to protect cell membranes from oxidative damage, the enzyme defense system, namely antioxidant enzymes including catalase (CAT), superoxide dismutase (SOD) and peroxidase (POD), removes excess reactive oxygen species, protects cells from free radical damage and reduces the degree of membrane lipid peroxidation (Pauls and Thompson, 1980; Gao and Zhang, 1998; Kholová et al., 2011). At the same time, under conditions of insufficient or excessive soil moisture, some osmotic regulatory substances including proline (Pro), soluble protein (SP), and soluble sugar (SS), are accumulated to maintain osmotic potential so that plants can obtain the water needed, ensure normal growth and improve their resilience to adversity (Manivannan et al., 2008). Plant endogenous hormones, chemical signaling substances produced in some cells or tissues (Tiwari et al., 2017), can regulate the physiological processes of other cells by interacting with specific protein receptors. Among these hormones, abscisic acid (ABA), indole-3-acetic acid (IAA), gibberellin (GA) and cytokinin (CTK) play important roles in regulating plant root development under water stress (Tiwari et al., 2017).

In this experiment, we selected *K. hirsuta* as the experimental material and measured the biomass, root-shoot ratio, root architecture parameters and physiological indexes to reveal changes of root architecture and root physiology at 10%, 25% and 40% moisture levels. To quantify root morphological and physiological indicators of root system development under different moisture levels, artificial neural network (ANN) model analysis was used to evaluate the importance of root architecture, physiological indexes and field water capacity in determining root biomass. This analysis revealed the regulatory mechanism of root adaptation to soil moisture changes at the seedling stage and explored the sand-growing characteristics of *K. hirsuta* to lay a foundation for resource development and variety breeding of pioneer grass species.

## 2 Materials and methods

### 2.1 Materials

*Kengyilia hirsuta* which grows in high altitude and alpine desert areas has developed a ‘rhizosheath-root system’ as a xero-phytic adaptive-trait. It is a plant species of genus *Kengyilia* Yen et J. L. Yang, and no other varieties have been found or cultivated at present. The seeds and sandy soil for the test were collected by the forage grass team of Qinghai-Tibet Plateau Research Institute of Southwest Minzu University in the sandy grassland in Waqie Town, Hongyuan County, Tibetan Qiang Autonomous Prefecture of Ngawa, Sichuan Province. The temperature difference between day and night in the sandy land in this area is large in summer. The sand surface is directly exposed to the sun during the day, and its temperature can reach more than 50°C, while can be as low as about 5°C at night and it has been enclosed in the past five years.

### 2.2 Experimental design

Three soil moisture gradients were set up by potted water control method, which were 10%, 25% and 40% field capacity, respectively. These soil moisture gradients are set for research, because the soil moisture of sandy grassland in Hongyuan county is about 3% ~ 8% and through pre-test screening, the phenotypes of *K. hirsuta* under these three water gradients are quite different.

Based on our experiment design, the completely randomized block design method has been chosen. The seedlings with identical growth were randomly divided into three groups. There were 48 replicates in each group and one seedling in each replicate. Meanwhile, each group was treated with 10%, 25% and 40% field capacity respectively, and other conditions were kept consistent. The specific steps were as follows:

The seeds were sterilized with 75% ethanol for 1min, washed for 3 times with distilled water, soaked in distilled water for 8h, and then washed for 3 times. Then, seeds of the same size and full size were spread in the Petri dish and were cultivated in an artificial climate box at a day/night temperature of 24/20°C with a photoperiod of 14/10 h light/dark, illumination of 30000 lx, and relative humidity ranging from 50 to 60%. After growing for 3 cm, seedings with the same growth were selected and transplanted into the PS nutrient bowl filled with the tested sandy soil. Each pot with one plant is poured with soil moisture (100% FC) and placed in the artificial climate box with the same culture condition as above. The seedling pot position was randomly changed every two days during the seedling culture period. Irrigate thoroughly on the 5th day after transplanting, and stop watering afterwards. When the soil moisture in all nutrient pots has dropped to 10% field capacity, seedlings were randomly divided into three groups, then weighed and watered according to the three designed moisture levels. The balance was used to weigh and then water was added to the set value at the same time every morning (10:00). Samples were taken when the plants grew to the three leaf stage.

### 2.3 Determination and measurement

After being loaded into the centrifugal tube, the roots were quickly put into the liquid nitrogen barrel, and then stored at -80°C to measure activity of antioxidant enzymes, MDA content, free Pro content, SP content and hormones content.

#### 2.3.1 Measurement of biomass

The whole plants were taken out from the nutrition bowl, then the roots were washed with distilled water and packed in envelopes. Afterward, samples were put into the oven at 110°C for 10 min and dried at 75°C until constant weight to measure abovground and root dry weight. Root-root shoot ratio was calculated as the ratio of underground dry weight to overground dry weight (Agathokleous et al., 2019).

#### 2.3.2 Measurement of root morphology

Root morphology was determined according to Liu et al. (2019). The roots in the transparent tray were scanned using a root scanner (EPSON Expression 12000XL) at a pixel of 600 dpi. Then the root analysis software (Win RHIZO Pro 2017A) was used to calculate and analyze the morphological indexes of the scanned original image, and the morphological indexes such as total root length, total root surface area, total root volume, average root diameter, root link average length, number of root tips and number of forks were obtained.

#### 2.3.3 Calculation of root fractal dimension and fractal abundance

Root analysis software (Win RHIZO Pro 2017A) was used to analyze the original image scanned by the digital scanner (EPSON Expression 12000XL), and the small square with side length r on the root distribution map and the number of small squares cut by the root, Nr, were obtained. The graph was drawn with logr and logNr as horizontal and vertical coordinates respectively. The equation of the regression line was as follows (Ketipearachchi and Tatsumi, 2000):


logNr=-FDlogr+logK

In this formula, K is the constant value. The negative number of the slope of the regression line is the fractal dimension FD and logK is the fractal abundance.

#### 2.3.4 Calculation of root topological index

The root topological index (TI) was calculated as follows (Ma and Wang, 2020):


TI= lgA/lgM

In the above formula, A is the total number of internal connections of the longest root channel; M is the total number of all external connections of the root system. Both A and M values could be calculated in the root analysis software (Win RHIZO Pro 2017A).

#### 2.3.5 Determination of malondialdehyde (MDA) content and relative conductivity (RC)

MDA content was assayed using Thiobarbituric acid reaction according to Draper and Hadley (1990). The 0.2 g sample and 1.6 ml 10% trichloroacetic acid solution were mixed and ground into pulp, centrifuged at 8000 g at 4°C for 10 min, and placed on ice for testing. After mixing 1.5 ml supernatant and 1.5 ml thiobarbituric acid solution, the mixture was placed in a 95°C water bath for 30 min, cooled in an ice bath, and centrifuged at 10000g for 10 min. The absorption (OD) values at 532 nm and 600 nm were measured. MDA content was determined according to fresh weight.

RC was measured by electrolyte extravasation volume method according to Luo et al. (2014). 0.2 g of fresh root was taken from each treatment and 4 ml of deionized water was added. After 24 h at room temperature, the conductivity of the solution R1 was measured. Then, the solution was boiled in a constant temperature water bath at 100°C for 20 min, and the solution conductivity R2 was measured after cooling to room temperature with cold water. The RC was calculated as follows:


RC (%)= R1/ R2 ×100%

#### 2.3.6 Determination of free proline (Pro) content and soluble protein content (SP)

Free Pro contwas determined by sulfosalicylic acid method reported by Zhang and Nie (2008). The 0.2g sample was mixed with 5mL 3% sulfosalicylic acid solution, extracted in a boiling water bath for 10 min, and then filtered after cooling. After being added 2 ml of filtrate, 2 ml of glacial acetic acid, and 2 ml of ninhydrin acid, the mixture was heated in a boiling water bath. After cooling, 4 ml toluene was added to the sample, shaken for 30 s, left for a while, 10 ml of the upper solution was taken and centrifuged at 3000g for 5 min. After that, the upper Pro red toluene solution was absorbed into the colorimetric cup, toluene was used as the blank control, OD value was measured at 520nm wavelength on the spectrophotometer, and Pro standard curve was made. SP content was estimated following the method of Coomassie Brilliant Blue according to Bradford (1976). 0.1 g sample was added with 1 ml PBS buffer solution, ground and homogenized, centrifuged at 8000g for 10 min. 1 ml supernatant was placed in a centrifuge tube, then 5ml Coomash bright blue solution was add. After mixing, the OD value at 620 nm was determined. The SP content was determined by the standard curve.

#### 2.3.7 Determination of antioxidant enzymes

Before the enzyme activity was measured, 0.2 g sample was added with 1.6 ml phosphate buffered saline (PBS), ground and homogenized, centrifuged at 8000g for 10 min, and the supernatant was taken and placed on ice for testing.

Referring to the study of Giannopolitis and Ries (1977), the activity of superoxide dismutase (SOD) was measured by nitroblue tetrazolium (NBT) photochemical reduction. One unit of SOD was defined as the amount of enzyme that inhibited the photochemical reduction of nitroblue tetrazole by 50%, and the OD value was measured at 560 nm. The activity of peroxidase (POD) was assessed using guaiacol method, and the OD value was measured at 470 nm (Nahid and Abdollah, 2018). Catalase (CAT) activity was determined by the method of UV absorption method, and the initial OD values and 1 min later OD values were determined at 240 nm (Zhang et al., 2018). When CAT was added into the reaction system, the absorbance of the reaction solution would decrease with the reaction time. Therefore, the activity of CAT could be calculated according to the change rate of the absorbance of the reaction solution.

#### 2.3.8 Determination of hormones contents

0.1 g sample was added to 0.9 mL PBS with pH=7.2~7.4, and then ground into homogenate. The supernatant was collected from homogenate separated by centrifugation at 4°C at 3000g for 20 min. The contents of abscisic acid (ABA), indole-3-acetic acid (IAA), gibberellin (GA) and cytokinin (CTK) in roots of *K. hirsuta* were determined using the Enzyme-Linked ImmunoSorbent Assay (ELISA) kit following the instructions from the manufacturer (Jiangsu Meimian industrial Co., Ltd. Jiangsu, China). The OD value of each hormone were detected *via* enzyme-labeled instrument (Varioskan LUX, Thermo Fisher Scientific). Linear regression equations of ABA, IAA, GA and CTK standard curves were calculated by using the concentration and OD value of the standard material.

### 2.4 Data analysis

#### 2.4.1 Artificial neural network analysis

Artificial neural network, an algorithm-based mathematical model that repeatedly adjusts the network connected between a large number of internal nodes by imitating the information processing capacity of biological neurons (Bagheri et al., 2017), can identify the complex nonlinear relationship between input and output variables (Rahimikhoob, 2010; Rossi et al., 2019). Multilayer perceptron ANN (MLPANN) normally has three layers, including an input layer, a hidden layer with multiple neurons, and an output layer, and is capable of Clarifying the most cluttered information (Mateo et al., 2011; Biglarijoo et al., 2017). Therefore, this paper chose MLPANN to build a neural network model, and used three-layer network to describe the mapping between the input and the output. After training and comparative analysis, the effects of root structure, physiology and field capacity on root biomass were explored.

P_i_ was the input layer, which was the root architecture and physiological indexes of *K. hirsuta* at seedling stage.

The output of the hidden layer is:


$${\text{Y}}_{\text{i}}={\text{F}}_{\text{j}}(\sum_{i=1}^{M}w_{ij}x_{i})$$

Where Y_i_ is the hidden layer, w_ij_ is the connection weight between input layer i and hidden layer j, and x_i_ is the input value. F_j_, the nonlinear activation function which is developed to calculate the output value of the hidden layer, is the hyperbolic tangent function (tanh). The formula is as follows:


$${\text{F}}_{\text{j}}\left(\text{u}\right)=\frac{\sinh u}{\cosh u}=\frac{e^{u}-e^{-u}}{e^{u}+e^{-u}}$$

The value of the output layer is the root biomass. Then the output of the output layer unit is:


$${\text{T}}_{\text{k}}={\text{F}}_{\text{k}}(\sum_{i=1}^{M}w_{jk}x_{j})$$

Where T_k_ is the output layer, w_jk_ is the connection weight between hidden layer j and output layer k, and x_i_ is the input value. F_k_, the linear activation function which is developed to calculate the output value of the output layer, is the identity function. The output value is calculated as follow:


$${\text{F}}_{\text{k}}\left(\text{u}\right)=\text{u}$$

In order to eliminate the influence caused by the large difference in the order of magnitude of index values, the measured data are normalized. After normalization, the values of each factor are distributed between [-1, 1]. In this paper, the data normalization and the inverse normalization of the simulated values output by the neural network are completed by using SPSS 26.0 software. The formula for data standardization is:


$${\text{I}}_{i}=\frac{x_{i-u}}{x_{max}-x_{min}}$$

In this formula, I_i_ is the standardized value; x_i_ is the measured value of index i; x_min_ and x_max_ are the minimum and maximum values of index i samples. u is the average of all samples of this indicator.

#### 2.4.2 Result processing

Results were analyzed by one-way ANOVA and tested for significance of difference by Duncan multiple-range method, the statistical significance level was below 0.05. Bivariate Pearson correlation analysis was used to analyze the relationship between physiological and morphological indexes. The neural network model of the influence of root architecture indexes, physiological indexes and field capacity on biomass was established by Multilayer perceptron. The data in the figures and tables represented as the mean ± standard error of 4 repetitions in the test. Excel 2016 was employed for summarizing all statistics, SPSS 26.0 was used for data analysis and Origin 2018 was assigned to plotting.

## 3 Results

### 3.1 Biomass and root-shoot ratio characteristics of *K. hirsuta* at the seedling stage under different soil moisture conditions

**Figure 1:**
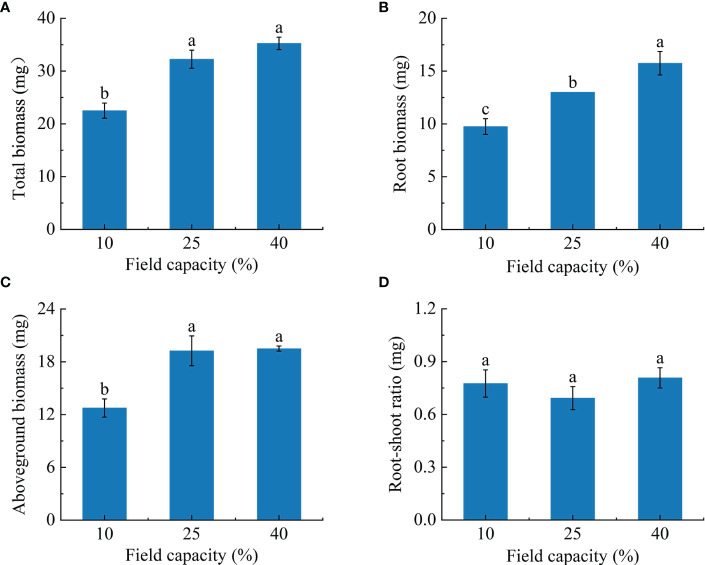
Effects of different soil moisture on total biomass **(A)**, root biomass **(B)**, aboveground biomass **(C)** and root-shoot ratio **(D)** of *K hirsuta.* Results were analyzed by one-way ANOVA and tested for significance of difference by Duncan multiple-range method. Each value represents the mean ± standard error. Each experiment was repeated four times. Different letters indicate that the differences between treatments were significant (*P<* 0.05).

With the increasing soil moisture gradient, the biomass of *K. hirsuta* at the seedling stage showed an upward trend (**Figure 1**). The total biomass and aboveground biomass of plants grown in soil under 25% and 40% field capacity were greater than those under 10% field capacity (*P*< 0.05) (**Figures 1A, C**), but no significant difference between 25% and 40% field capacity was observed in these data (*P* > 0.05). There were significant differences in root biomass among the 10%, 25% and 40% field capacity treatments (*P*< 0.05). Moreover, as field capacity increased, root biomass increased. The root biomasses in the 25% and 40% field capacity treatments were 1.3 and 1.6 times higher than that of the 10% field capacity treatment, respectively (**Figure 1B**). However, the root-shoot ratio showed no significant changes under different field capacities (*P* > 0.05) (**Figure 1D**).

### 3.2 Root morphology characteristics of *K. hirsuta* at the seedling stage under different soil moisture conditions

**Figure 2:**
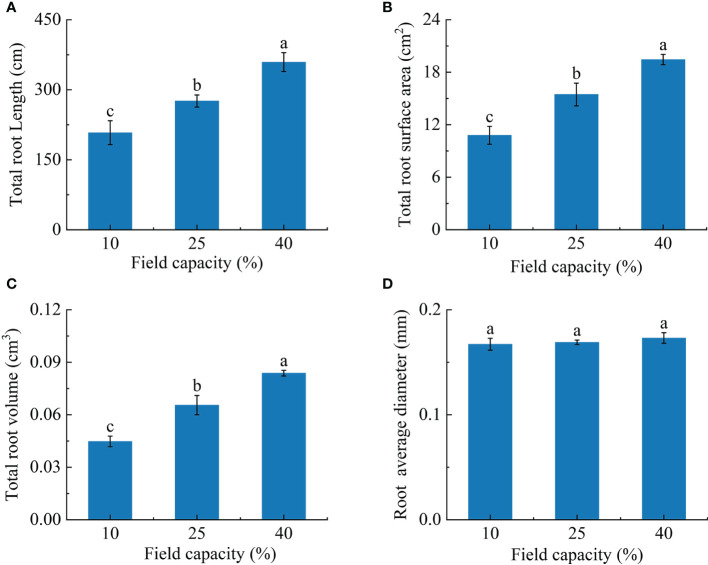
Effects of different soil moisture on total root length **(A)**, total root surface area **(B)**, total root volume **(C)** and average root diameter **(D)** of *K hirsuta*. Results were analyzed by one-way ANOVA and tested for significance of difference by Duncan multiple-range method. Each value represents the mean ± standard error. Each experiment was repeated four times. Different letters indicate that the differences between treatments were significant (*P*< 0.05).

Most root morphological indexes were sensitive to the different soil moisture conditions. The total root length, total root volume and total root surface area of plants grown in soil under the three field capacity treatments showed remarkable differences (*P*< 0.05). The total root length, total root volume and total root surface area increased with increasing soil moisture. Total root length in the 25% and 40% field capacity treatments was 1.3 and 1.7 times than that in the 10% field capacity treatment, respectively (**Figure 2A**), total root surface area was 1.5 and 1.9 times greater, respectively (**Figure 2B**), and total root volume was 1.3 and 1.9 times greater, respectively (**Figure 2C**). However, no notable differences were observed for average root diameter (*P* > 0.05) (**Figure 2D**).

**Figure 3:**
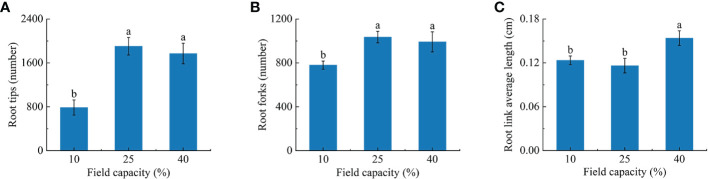
Effects of different soil moisture on root tips **(A)**, root forks **(B)**, root link average length **(C)** of *K hirsuta.* Results were analyzed by one-way ANOVA and tested for significance of difference by Duncan multiple-range method. Each value represents the mean ± standard error. Each experiment was repeated four times. Different letters indicate that the differences between treatments were significant (*P*< 0.05).

As the soil moisture rose, the number of root tips and forks first increased and then decreased. The numbers of root tips and forks under 10% field capacity were significantly lower than those under 25% and 40% field capacity (*P<* 0.05). The numbers of root tips and forks under 25% field capacity were 2.4 and 2.3 times those under 10% field capacity, and 1.3 and 1.2 times those under 40% field capacity, respectively (**Figures 3A, B**). The root link average length under 40% field capacity was significantly longer than those under 25% and 10% (*P*< 0.05) (**Figure 3C**).

### 3.3 Root fractal dimension, fractal abundance and root topological index characteristics of *K. hirsuta* at the seedling stage under different soil moisture conditions

There were no marked differences in the root fractal dimension (*P >*0.05) (**Table 1**). As can be seen from the table, the root fractal abundances of the 25% and 40% field capacity treatments were greater than that of the 10% field capacity treatment (*P*<0.05), but there were no significant differences between the 25% and 40% field capacity treatments (*P >*0.05).

**Table 1:** Effects of different soil moisture on root fractal dimension, root fractal abundance and root topological index of *K. hirsuta*.

Field capacity (%)	Root fractal dimension	Root fractal abundance	Root topological index
10%	1.263 ± 0.045a	3.323 ± 0.110b	0.819 ± 0.053a
25%	1.229 ± 0.019a	3.557 ± 0.050a	0.762 ± 0.019b
40%	1.2495 ± 0.015a	3.638 ± 0.051a	0.755 ± 0.013b

Results were analyzed by one-way ANOVA and tested for significance of difference by Duncan multiple-range method. Each value represents the mean ± standard error. Each experiment was repeated four times. Different letters indicate that the differences between treatments were significant (P< 0.05).

As the soil moisture rose, the root topological index of *K. hirsuta* decreased continuously (**Table 1**). The topological index under 10% field capacity was significantly higher than those under 25% and 40%, which were closer to 1 (*P<*0.05). This result indicated that the root branching pattern changed from dichotomous branching to herringbone branching with the decrease of soil moisture.

### 3.4 Membrane permeability and osmotic regulatory substances characteristics in *K. hirsuta* roots at the seedling stage under different soil moisture conditions

**Figure 4:**
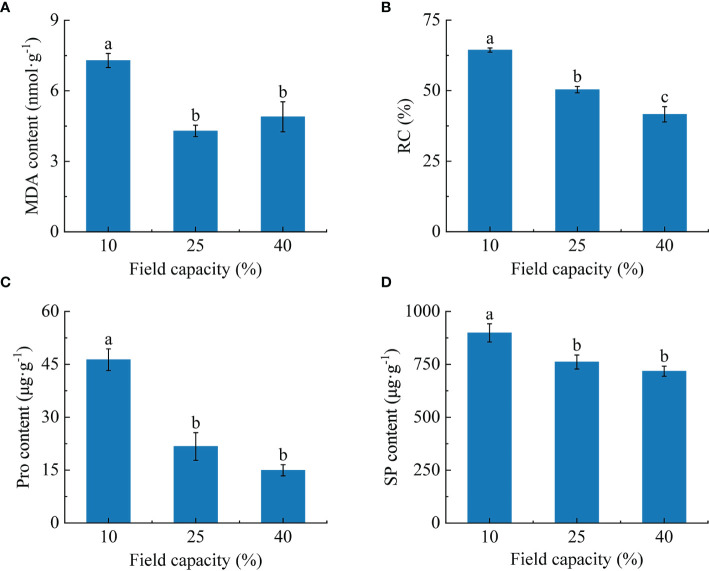
Effects of different soil moisture on malondialdehyde (MDA) content **(A)**, relative conductivity (RC) **(B)**, proline (Pro) content **(C)** and soluble protein (SP) content **(D)** of *K hirsuta.* Results were analyzed by one-way ANOVA and tested for significance of difference by Duncan multiple-range method. Each value represents the mean ± standard error. Each experiment was repeated four times. Different letters indicate that the differences between treatments were significant (*P*< 0.05).

The investigation showed that the MDA content and RC of roots varied with different soil moisture treatments (**Figure 4**). The MDA content of roots under 10% field capacity was significantly higher than those under 25% and 40% (*P<* 0.05), at 1.7 and 1.5 times, respectively. Additionally, there were significant differences in RC among the three field capacity treatments (*P*< 0.05). The RC displayed a declining trend with the increase of soil moisture, being lowest in the 40% field capacity treatment (**Figure 4B**).

With the increase of soil moisture, the free Pro and SP contents decreased (**Figure 4**). The free Pro and SP contents of roots under 10% field capacity were notably higher than those under 25% and 40% field capacity (*P<* 0.05), but no significant differences were found between the 25% and 40% field capacity treatments (*P >*0.05).

### 3.5 Antioxidant enzyme activities characteristics in *K. hirsuta* roots at the seedling stage under different soil moisture conditions

**Figure 5:**
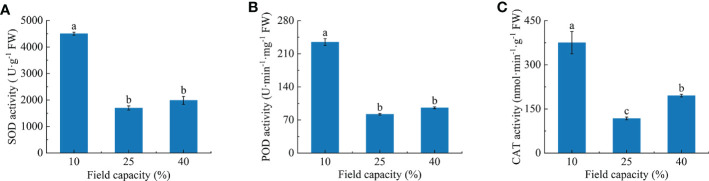
Effects of different soil moisture on superoxide dismutase (SOD) **(A)**, peroxidase (POD) **(B)** and catalase (CAT) **(C)** activities of *K hirsuta*. Results were analyzed by one-way ANOVA and tested for significance of difference by Duncan multiple-range method. Each value represents the mean ± standard error. Each experiment was repeated four times. Different letters indicate that the differences between treatments were significant (*P*< 0.05).

With the increasing soil moisture, the activities of root antioxidant enzymes first decreased and then increased slightly (**Figure 5**). The SOD and POD activities at 10% field capacity were significantly higher than those at 25% and 40% field capacity (*P*< 0.05). Compared with 25% and 40% field capacity, SOD activity was increased by 165.0% and 126.6% (**Figure 5A**), and POD activity was increased by 186.0% and 145.1% (**Figure 5B**), respectively, under 10% field capacity. Notable differences were observed for CAT activity among the three field capacity treatments (*P*< 0.05) and CAT activity under 10% field capacity was increased by 218.8% and 92.1% compared with 25% and 40% field capacity, respectively (**Figure 5C**).

### 3.6 Hormones contents in *K. hirsuta* roots at the seedling stage under different soil moisture conditions

**Figure 6:**
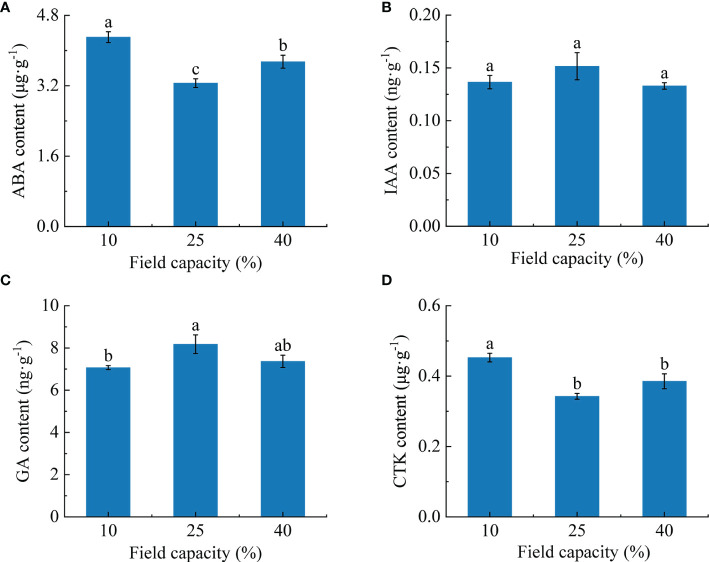
Effects of different soil moisture on abscisic acid (ABA) **(A)**, indole-3-acetic acid (IAA) **(B)**, gibberellin (GA) **(C)** and cytokinin (CTK) **(D)** contents of *K hirsuta.* Results were analyzed by one-way ANOVA and tested for significance of difference by Duncan multiple-range method. Each value represents the mean ± standard error. Each experiment was repeated four times. Different letters indicate that the differences between treatments were significant (*P*< 0.05).

Root hormone contents varied under the different soil moisture treatments (**Figure 6**). With the increase of soil moisture, the ABA content showed a significant trend of an initial decrease followed by an increase (*P<* 0.05), and was lowest under 25% field capacity (**Figure 6A**). The IAA content in roots was not obviously affected by the different soil moisture treatments (**Figure 6B**), and the differences between treatments did not reach a significant level (*P* > 0.05). With the increase of soil moisture, the GA content showed a trend of a significant increase at first and then a slight decline (**Figure 6C**). The GA content was highest under 25% field capacity and was significantly higher than under 10% (*P*< 0.05). The CTK content was at its maximum under 10% field capacity and was significantly higher than under 25% and 40% (*P<* 0.05) (**Figure 6D**).

### 3.7 Correlation analysis of root physiology and root architecture in *K. hirsuta* at the seedling stage under different soil moisture conditions

**Figure 7:**
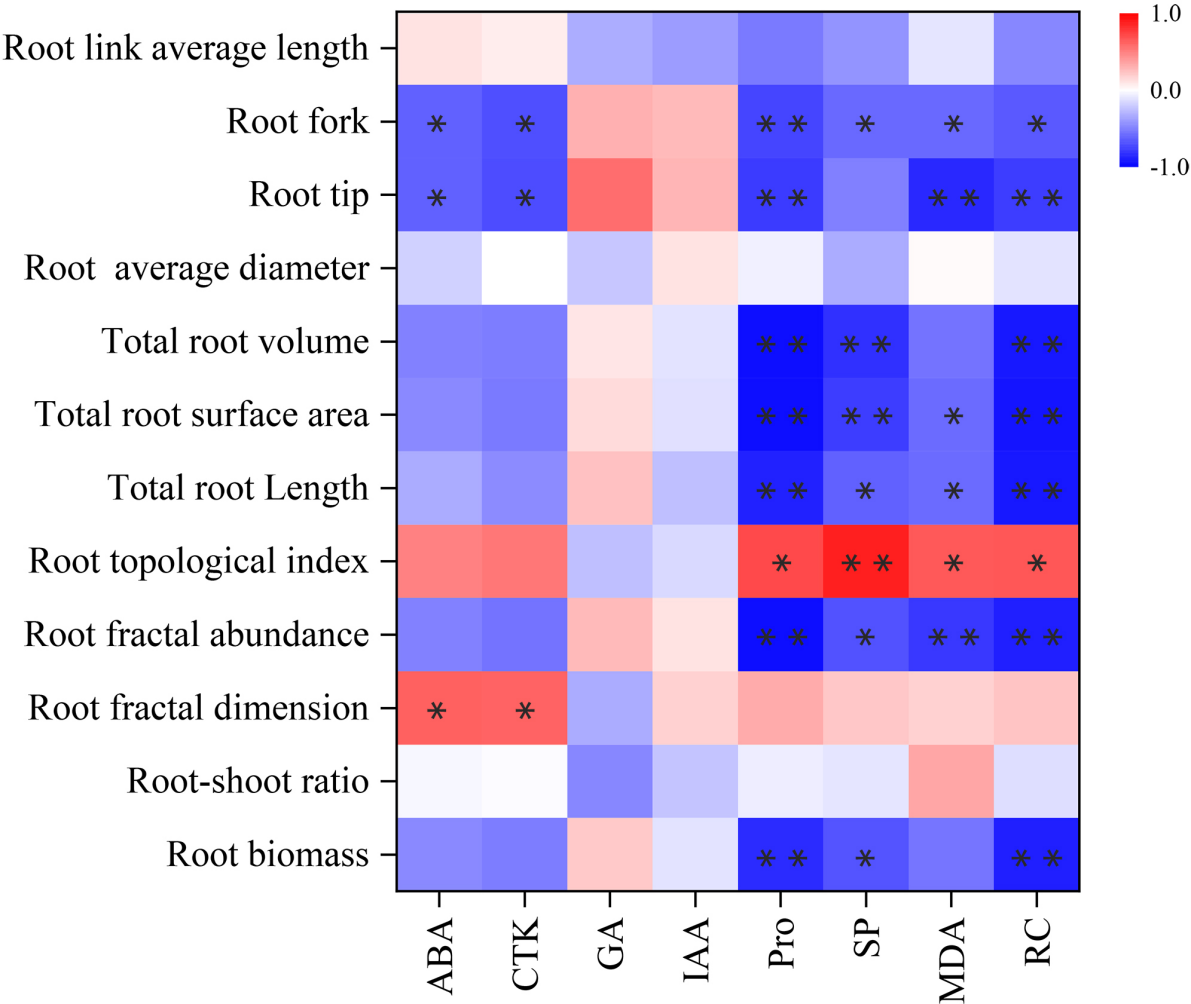
Correlation between root physiology and architecture. Each experiment was repeated four times. Significant correlation between root physiology and architecture are indicated by one asterisk (* *P*<0.05) or two asterisk (** *P*<0.01), as determined by Bivariate Pearson correlation analysis.

Correlation analysis was performed on root physiology and root architecture (**Figure 7**). The results showed that ABA and CTK had significant positive correlation with fractal dimension (*P<* 0.05), and significant negative correlation with root tips and forks (*P<* 0.05). Pro showed a significant positive correlation with topological index (*P<* 0.05), and very significant negative correlation with root biomass, root fractal abundance, total root length, total root volume, root tips and root forks (*P<* 0.05). There was a very significant positive correlation between SP and topological index (*P<* 0.01), significant negative correlation with root biomass, fractal abundance, total root length and forks (*P<* 0.05), and very significant negative correlation with total root surface area and total root volume (*P*< 0.01). MDA had a significant positive correlation with topological index (*P<* 0.05), significant negative correlation with total root length, total root surface area and root forks (*P<* 0.05), and extremely significant negative correlation with fractal abundance and root tips (*P*< 0.01). RC was positively correlated with topological index (*P<* 0.05), negatively correlated with root forks (*P<* 0.05), and extremely significant negatively correlated with fractal abundance, total root length, total root surface area, total root volume and root tips (*P*< 0.01). There were only weak correlations between root morphological indexes and GA or IAA (*P* > 0.05).

### 3.8 Artificial neural network analysis

The artificial neural network model we established included an input layer, hidden layer and output layer, in which root architecture indexes (including root fractal dimension, root fractal abundance, root topological index, total root length, total root surface area, total root volume, root average diameter, root tips, root forks, and root link average length), root physiology indexes (including MDA, RC, SOD, POD, CAT, Pro, SP, ABA, IAA, GA and CTK) and the three field capacity treatments (10%, 25% and 40%) were used as input variables (a total of 24 values), and the hidden layer was set as 1 layer. After several network tests and adjustments, seven nodes in the hidden layer were determined to give the best estimation. Subsequently, root biomass was used as the output layer. Finally, the predictive value, relative error and importance of independent variables of the neural network were determined.

**Figure 8:**
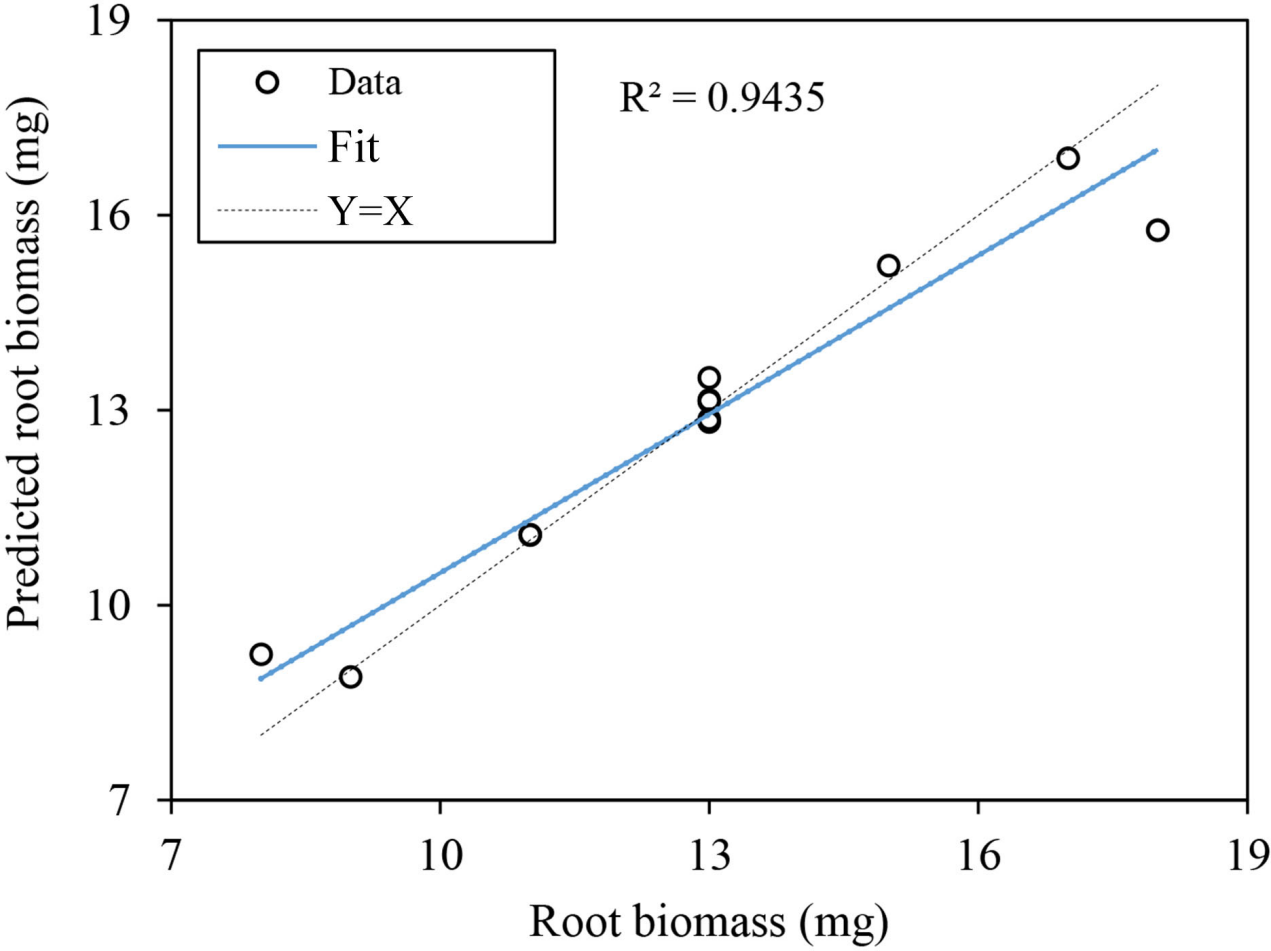
Relationship between values of root biomass as measured and predicted by the ANN model.

The predicted data versus the observed data were plotted for the testing data set, and the coefficients of determination (*R* ^2^) were determined (**Figure 8**). The validation analysis showed that the ANN models gave a high *R* ^2^ and low relative error, which were 0.9435 and 0.4%, respectively, indicating that the network model fitted the original data well and had high simulation accuracy, and could be used to analyze the factor importance of other indexes on root biomass.

**Figure 9:**
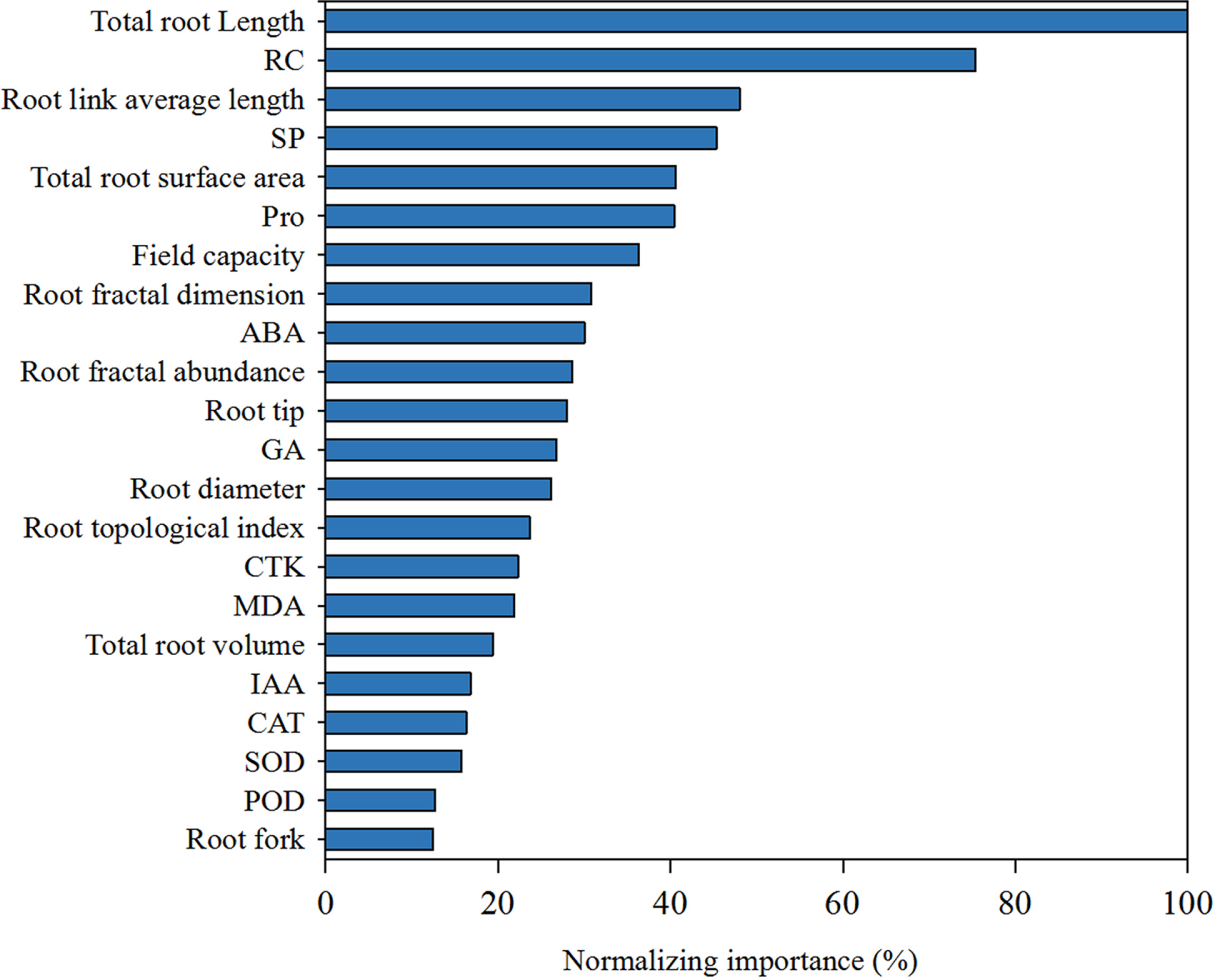
The factor importance of root architecture indexes, physiological indexes and Field capacity to root biomass at seedling stage of *K. hirsuta*.

In the results, the indices with higher normalized importance values could explain the variation of the overall development components of *K. hirsuta*. As can be seen from **Figure 9**, total root length was identified as the most important factor affecting overall root development and showed the greatest normalized importance (100%). Other important factors included RC, root link average length, SP, total root surface area, field capacity and Pro, with normalized importance values in ranked order of 75.4%, 48%, 45.3%, 40.6%, 40.4%, 36.3%, respectively.

## 4 Discussion

### 4.1 Effects of different soil moisture conditions on biomass and root-shoot ratio

The biomass of plants is the product of photosynthesis and thus reflects the ability of plants to utilize external nutrients, so it is an important indicator of plant adaptation to water change Hester et al. (2001); Guo et al. (2016) found a significant correlation between biomass and soil moisture in various plants, among which *Tamarix ramosissima* showed a significant positive correlation and *Haloxylon ammodendron* displayed a significant negative correlation with soil moisture. Dalal et al. (2018) reported that the root biomass of wheat increased in response to drought stress, while Sofi et al. (2018) reported that root biomass showed a downward trend when *Phaseolus vulgaris* L. was subjected to drought stress. Consistent with the results of Sofi, our research revealed that total biomass, root biomass and aboveground biomass were significantly lower under 10% field capacity than under 25% and 40%. These results suggested that the growth and development of *K. hirsuta* was restricted under the 10% field capacity condition, which represents an arid environment, and the photosynthetic capacity of seedlings was weakened, resulting in a decrease of photosynthetic product, while field capacities of 25% and 40% were beneficial to the survival of *K. hirsuta*, and thus increased biomass accumulation (Rahman et al., 2020).

The root-shoot ratio directly reflects the distribution of aboveground and root biomass (Enquist and Niklas, 2002). According to many studies, when a plant encounters drought stress, the substance distribution changes and the amount of assimilates transported from aboveground parts to the roots increases, which makes the root-shoot ratio rise as the plant seeks more water (Smucker and Aiken, 1992). It was reported that the root-shoot ratio of maize at different growth stages increased with increasing degrees of drought (Benjamin et al., 2014). Nevertheless, there were no obvious changes in our study when *K. hirsuta* was exposed to different soil moisture contents. This might have been because *K. hirsuta* is a drought-tolerant species, and the root-shoot ratio is controlled by its own genetic mechanism to adapt to a wider range of soil moisture levels, such that changes of field capacity in the range of 10%–40% had no significant effect on the root-shoot ratio (Tang et al., 2020).

### 4.2 Changes of root architecture under different soil moisture conditions

Root morphology plays an important role in root absorption and utilization of water and nutrients (Lynch, 1995), and can respond to changes of soil moisture to a certain extent (Comas et al., 2013). Some previous studies suggested that when soil moisture was insufficient, the total root length, total root surface area and root diameter of plants increased, which was conducive to the large-scale absorption of soil water and nutrients (Mu et al., 2005), while others had found that drought stress inhibits root growth and development (David, 1991). This discrepancy may be related to plant growth stages and tolerance to water stress. In this study, the total root length, total root volume, total root surface area and root link average length increased as the field capacity rose from 10% to 40%. This is consistent with the finding of Liu et al. (2019), who showed that when soil moisture reduced, root length, root surface area, root volume and other indicators of switchgrass decreased significantly. Our experimental results also showed that root tips and root forks were increased under 25% and 40% field capacity compared with 10%. Scholars have shown that root length and root link average length reflect root elongation and water absorption efficiency (Mu et al., 2005; Tang et al., 2020), as the root tips is one of the main channels for water and nutrients to enter the plant (Ma et al., 2011), root forks reflect the complexity of root architecture (Tang et al., 2020). Therefore, considering the observations reported above, when field capacity was too low, at 10%, which was beyond the adaptive range of *K. hirsuta*, root growth was inhibited, while an increase of soil moisture could promote the root development of *K. hirsuta* at the seedling stage.

Root fractal characteristics and the topological index respond to changes of soil moisture. A higher fractal dimension represents a higher degree of root development (Oppelt et al., 2001), larger fractal abundance indicates a wider distribution range of roots in the soil and better resource absorption efficiency (Shan et al., 2012), and the topological index determines the root spatial distribution state and affects the acquisition of resources in the soil environment (Berntson, 1997). Chen et al. (2006) found that the fractal dimension of *Tilia tomentosa* decreased under waterlogging or drought conditions and increased under suitable moisture conditions, but there was no significant change in the fractal dimension of *K. hirsuta* under the three soil moisture conditions in our research, which may be because the fractal dimension of different plants responds differently to soil moisture. Shan et al. (2012) reported that with an increase in the degree of drought, the fractal abundance of *Reaumuria soongorica* became smaller. Similarly, the root fractal abundance of *K. hirsuta* under 10% field capacity conditions was remarkably lower than under the 25% and 40% field capacity treatments and suggested that *K. hirsuta* at the seedling stage adapted to drought stress by reducing the root expansion range. In previous research, wheat roots were deeply rooted under limited water conditions with a herringbone branching pattern, but were shallow rooted under waterlogging conditions with a dichotomous branching mode (Miao et al., 2019). Our results were similar to those of the above research that the root topological index of *K. hirsuta* under 10% field capacity conditions was significantly larger than those under 25% and 40%, which were closer to herringbone branching. This indicated that the root branching habit becomes closer to herringbone branching by reducing overlapping roots and increasing link length, so as to reduce the competition for nutrients within the root system and ensure effective nutrient distribution for *K. hirsuta* to adapt to an arid environment (Shan et al., 2012).

### 4.3 Responses of physiological parameters to different soil moisture conditions

Differences of soil moisture lead to a series of physiological metabolism changes in roots, including changes of membrane structure and function, the activation of the protective enzyme system, osmotic regulation, and hormone contents (Farooq et al., 2009).

The MDA content and RC can indicate the degree of stress injury to plants under water stress (Zhao et al., 2011). We found that the MDA content and RC in *K. hirsuta* roots were highest under 10% field capacity conditions, and were significantly higher than under 25% and 40%, consistent with the results of previous studies. Additionally, MDA and RC were negatively correlated with fractal abundance, total root length, total root surface area, root tips and forks. This suggested that root cell membranes were damaged and their permeability increased when *K. hirsuta* was grown in soil with 10% field capacity, which inhibited the growth and development of the root system and was not conducive to its establishment.

Pro and SP are key osmotic regulatory substances in plants that can maintain osmotic pressure and alleviate the threat of oxidative stress during drought stress (Babita et al., 2010; Ben et al., 2014). It was demonstrated that Pro and SP contents in *P. cornutum* roots at the seedling stage increased with the severity of drought under mild and moderate water stress Zhang et al. (2016). Thus, it was shown that under mild and moderate water stress, plants could maintain low intracellular osmotic potential by increasing their Pro and SP contents, improve the ability of cells to retain water, and reduce the damage caused by drought in *P. cornutum*, but severe stress damages root cells and makes plants unable to effectively maintain osmotic pressure. In our study, the Pro and SP contents increased as soil moisture declined at the seedling stage in *K. hirsuta* and reached their highest values at 25% field capacity. The Pro and SP contents were positively correlated with the root topological index, and negatively correlated with root biomass, fractal abundance, total root length, total root surface area, total root volume, root tips and root forks. In general, this was because the root Pro and SP contents of *K. hirsuta* at the seedling stage were sensitive to water deficit and under drought conditions the root system maintained cell turgor by increasing the contents of free Pro and SP, which restricted the development of root morphology, so as to maintain the original physiological activities and adapt to water change.

Lack of soil moisture can lead to the accumulation of reactive oxygen species in plants, but plants can remove excess reactive oxygen species by increasing the activities of SOD, POD and CAT in the enzyme antioxidant defense system (Mittler, 2002). In a study by Zhang et al. (2016), the root antioxidant enzyme activity of *Pugionium cornutum* at the seedling stage increased with an increasing degree of drought, and the activities of SOD, POD and CAT in roots showed an upward trend. Differing from the above study, the SOD, POD and CAT activities here showed a trend of first decreasing and then increasing with an increasing moisture gradient, and were significantly higher at 10% field capacity than at 25% and 40%, with the lowest activities at 25% field capacity. Therefore, under the stress of 10% field capacity, the degree of membrane peroxidation in *K. hirsuta* roots was more serious, which triggered the antioxidant system in roots and improved the activity of antioxidant enzymes, while under the 25% field capacity condition, the antioxidant enzyme activity in *K. hirsuta* roots was lowest, and the metabolism of reactive oxygen species was in equilibrium (Kholová et al., 2011).

The root development of plants is closely related to their resistance to environmental drought stress, and root growth depends on hormone regulation (Guo et al., 2018; Ye et al., 2018). Among the many plant hormones, growth regulators such as ABA, IAA, GA and CTK play important regulatory roles in plant root responses to soil water changes (Tiwari et al., 2017).

ABA is produced in roots and an increase of ABA content can promote the expression and activity of aquaporins, and enhance the elongation of the root cell wall to promote root water absorption under water deficit (Lutz and Wolfgang, 2005; Guo et al., 2018). Ye et al. (2018) found that in an arid environment, the ABA content of maize increased by large quantities to enhance the drought resistance of plants, which was similar to the results of Ding et al. (2016) who reported that PEG treatment for 24 h induced ABA accumulation in the roots of rice seedlings. In our experiment, the ABA content first decreased and then increased with the rise of soil moisture, and was the highest under 10% field capacity conditions. We also found ABA in *K. hirsuta* roots was significantly positively correlated with the fractal dimension and notably negatively correlated with root tips and forks. The above results showed that the root system of *K. hirsuta* synthesized a large amount of ABA to promote water absorption and reduce root tip and fork numbers to adjust the root architecture to adapt to water shortage conditions, while under suitable soil moisture conditions, ABA was present at a normal level.

IAA regulates the processes of principal growth, lateral root formation and elongation, and adventitious root and root hair development (Guo et al., 2018). In the absence of soil moisture, the IAA concentration and mode of transport change the root morphology and improve the adaptability of plants to adversity (Krome et al., 2010; Guo et al., 2018). Wang et al. (2019) reported that the synthesis of endogenous IAA in sweet potato roots was significantly inhibited by 10 days of drought stress, resulting in a decrease of IAA content. Unlike the above research results, Mahouachi et al. (2007) found that the IAA content in roots of papaya seedlings did not change under drought stress. Consistent with the results of Mahouachi, the IAA content in *K. hirsuta* roots was not sensitive to different water conditions, indicating that IAA in the roots of different plants has different responses to water.

GA is related to the growth of the plant root structure and the formation of lateral roots, and its content changes under water deficit stress (Fonouni-Farde et al., 2019). Studies have found that gene expression related to GA biosynthesis was upregulated in soybean (Bashir et al., 2019), but the endogenous GA content in pea roots decreased (Yan et al., 2009) under drought stress. The two different results above may be due to changes of GA content related to plant species. In this study, as the soil moisture rose, the GA content of *K. hirsuta* first increased and then declined, reaching its highest value under 25% field capacity conditions, which was significantly higher than that under 10% field capacity conditions. These results suggested that *K. hirsuta* roots coped with water deficiency conditions by reducing GA metabolism but maintained a high GA content under moderate and moist soil moisture conditions to promote root development.

CTK, mainly synthesized in roots, regulates root microtubule tissue development, root cell extension, root geotropism, adventitious root differentiation and lateral root development (Aloni et al., 2004; Wei et al., 2012; Guo et al., 2018). It was reported that the CTK content in sorghum roots and leaves decreased significantly under drought stress (Zhou et al., 2014). However, Gong et al. (2018) found that the CTK content in seedling roots of two wheat varieties increased significantly under water-limited conditions, and was significantly negatively correlated with total root length and total root surface area, which was consistent with the results of our experiment. The CTK contents in *K. hirsuta* roots growing in soil under 25% and 40% field capacity conditions were remarkably lower than in those growing at 10% field capacity, and were significantly negatively correlated with root tips and root forks. This may be because under conditions of moderate and sufficient water, the CTK content in *K. hirsuta* roots decreased, which promotes better root development.

### 4.4 Based on the actual demand for soil moisture of *K. hirsuta*, the optimal growth conditions of *K. hirsuta* were explored from the perspective of water economy

Water, the source and the foundation of life, is important part of life on the earth. With the global climate change and the interference of human activities, water resources are being affected and gradually scarce, which will bring many adverse effects, threatening human health and living environment (Falkenmark, 2013). At the same time, water is the most important natural resource in agricultural production, and irrigation is a common way of water demand for crop cultivation. Improper use of water and fertilizer will cause water waste and land salinization, which will harm agricultural production (Wege, 2022). Therefore, exploring the minimum optimum water demand of plants is beneficial to plant growth and water use. It was reported that plant varieties with strong stress resistance can help save water resources and cope with climate change (Hanjra and Qureshi, 2010). In our study, *K. hirsuta*, the wind proof and sand fixing plant, could be grown and cultured normally under 25% and 40% field capacity, and some indicators were developed better under 25% field capacity. Thus, in the actual cultivation, 25% field capacity conditions can be selected to *K. hirsuta*, which not only ensure the normal growth of plants, but also reduce the consumption of water resources, providing reference for the cultivation of other desertification plants.

### 4.5 Analysis of the importance of root architecture indexes, physiological indexes and field capacity for root biomass of *K. hirsuta* at the seedling stage

In previous studies, root biomass has been considered an important index to evaluate plant development, reflect the distribution of the root system and measure the overall growth and development of plant roots (Lv et al., 2019; Wu and Sheng, 2020; Su et al., 2021). The root architecture and physiological indexes of *K. hirsuta* we measured were numerous and complex, and it was difficult to show a linear relationship with root biomass. Therefore, we used artificial neural network analysis to explore and quantify the influence of various root system architecture indexes, physiological indexes and the field capacity gradient on root biomass variation under different soil moisture conditions. The neural network model we constructed showed high *R* ^2^ and low relative error, indicating that it had high simulation accuracy and was suitable to analyze the importance of factors affecting root biomass.

Among all the root architecture indexes of *K. hirsuta*, total root length was the most obvious factor causing variation of root biomass, and its normalized importance was 100%. However, root link average length and total root surface area also had an important impact on root biomass. Lv et al. (2019) established a root biomass model with high simulation accuracy using the least squares method and found that root length played the most important role in influencing maize root architecture and root biomass. In a study by Rong et al. (2020), the root biomass of *Fokienia hodginsii* seedlings was significantly positively correlated with total root length, total root surface area and root average diameter. The above results were consistent with our experiment. Among all root physiological indexes, RC, SP and Pro had significant effects on root biomass and combined with correlation analysis, our results suggested that these physiological indexes negatively regulated root development. The results of Du et al. (2017) showing that physiological indexes such as Pro and SP in barley negative regulated root development were similar to ours. Furthermore, the results of factor importance analysis and one-way ANOVA showed that different field capacities had a significant effect on root biomass. In the results discussed above, although the plant species, root types and habitats (water, climate, soil conditions) varied, the main factors affecting root development were similar, but whether there are differences in other plants remains to be determined.

## 5 Conclusion

We found that 25% and 40% field capacity conditions were beneficial to the growth and acquisition of nutrients and water of *K. hirsuta* roots at the seedling stage, with better development of root morphology and a smaller variation range of physiological indexes.

However, the root growth and development of *K. hirsuta* was inhibited under the 10% field capacity condition, which was reflected in the reduction of biomass accumulation and the obstruction of root morphological development. This suggested *K. hirsuta* adapts to an arid environment by simplifying its root configuration and reducing its root branches to make the root topology closer to herringbone branching. Additionally, the activity of antioxidant enzymes in roots was high to slow down the degree of membrane lipid peroxidation and membrane permeability, free Pro and SP contents were high, and more ABA and CTK were synthesized to resist drought stress.

We found that three root architecture indexes including total root length, root link average length and total root surface, three root physiological indexes including RC, SP and Pro, and the field capacity were key factors affecting root biomass and had an important impact on the growth and development of *K. hirsuta* roots at the seedling stage.

## Data availability statement

The raw data supporting the conclusions of this article will be made available by the authors, without undue reservation.

## Author contributions

XC, YC contributed to the study design; XC, YC, WZ, WLZ, HW, QZ, were involved in drafting the manuscript and agree to be accountable for the work. All authors read and approved the final manuscript.
